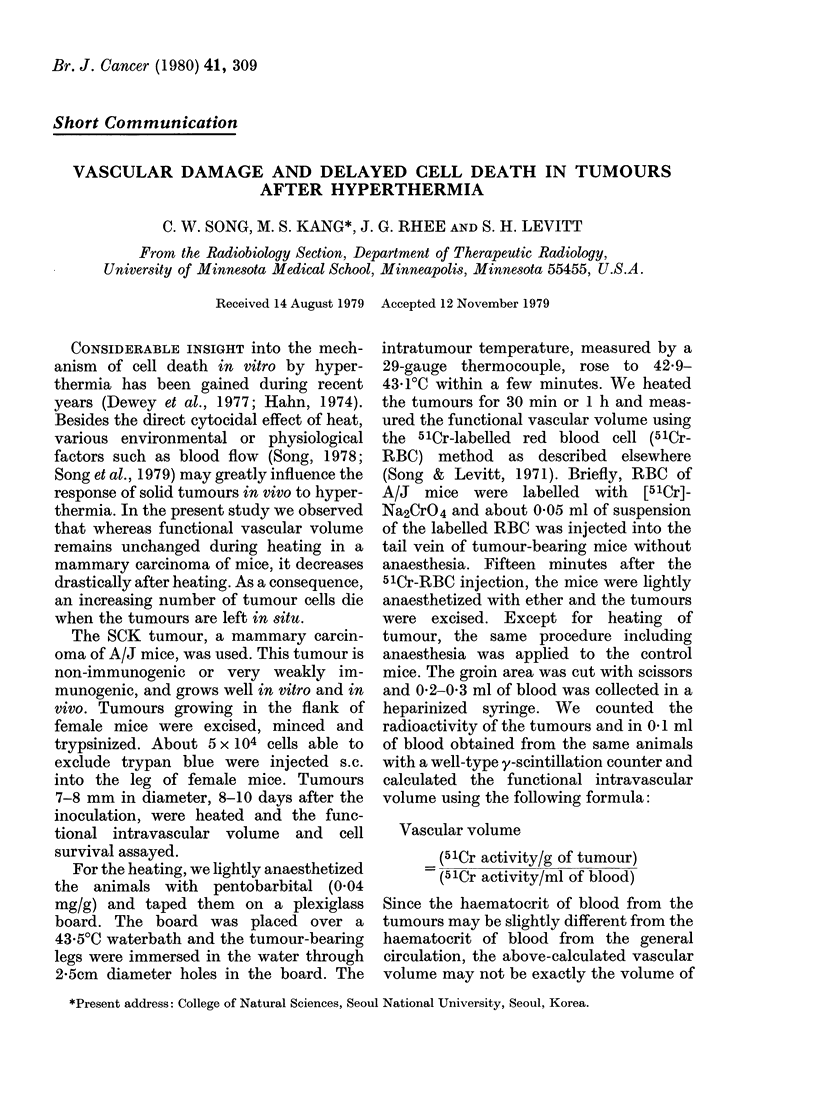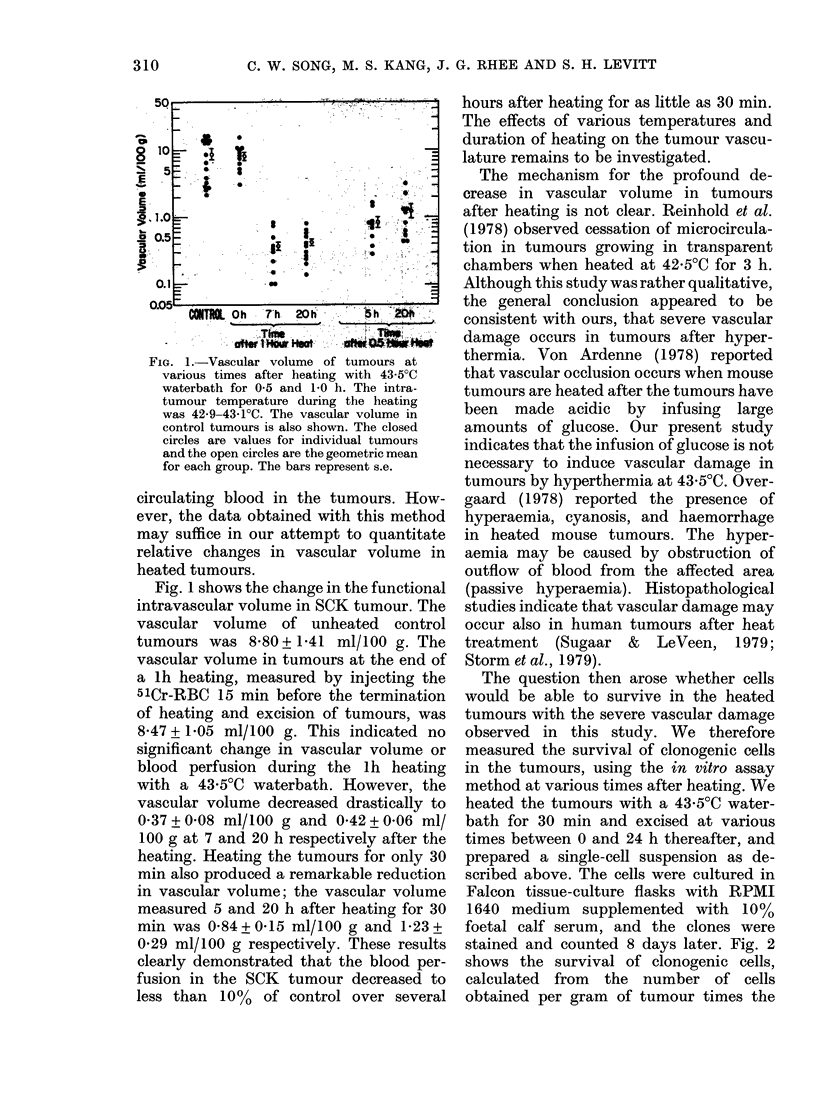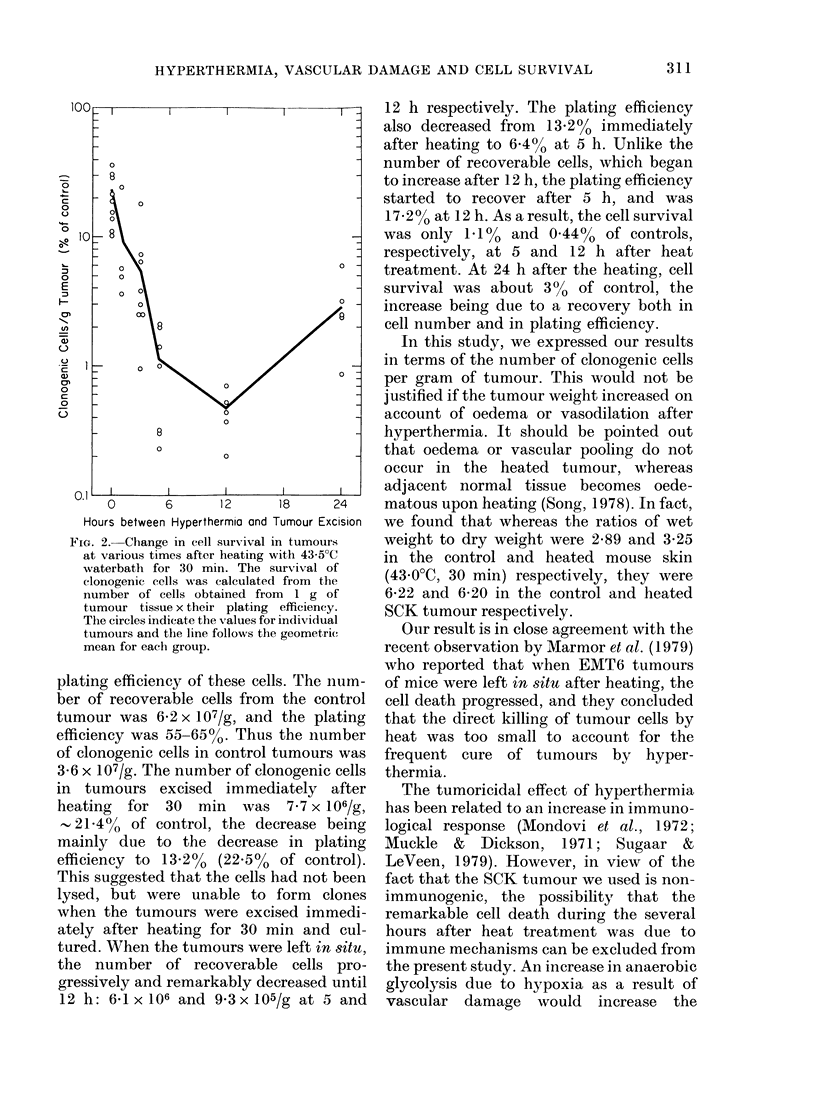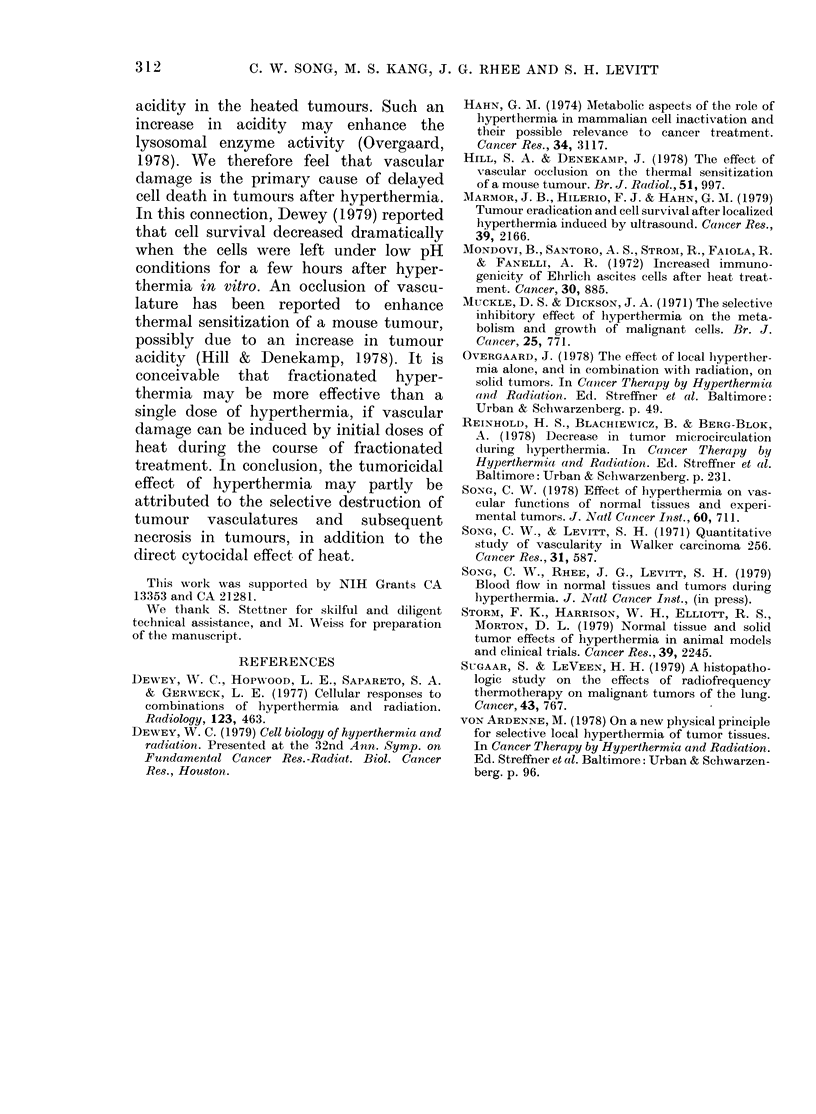# Vascular damage and delayed cell death in tumours after hyperthermia.

**DOI:** 10.1038/bjc.1980.45

**Published:** 1980-02

**Authors:** C. W. Song, M. S. Kang, J. G. Rhee, S. H. Levitt


*Br. J. Cancer* (1980) **41**, 309

***Short Communication***

### VASCULAR DAMAGE AND DELAYED CELL DEATH IN TUMOURS AFTER HYPERTHERMIA

C. W. SONG, M. S. KANG*, J. G. RHEE AND S. H. LEVITT

*From the Radiobiology Section, Department of Therapeutic Radiology, University of Minnesota Medical School, Minneapolis, Minnesota* 55455, *U.S.A.*

Received 14 August 1979 Accepted 12 November 1979

CONSIDERABLE INSIGHT into the mechanism of cell death *in vitro* by hyperthermia has been gained during recent years (Dewey *et al.*, 1977; Hahn, 1974). Besides the direct cytocidal effect of heat, various environmental or physiological factors such as blood flow (Song, 1978; Song *et al.*, 1979) may greatly influence the response of solid tumours *in vivo* to hyperthermia. In the present study we observed that whereas functional vascular volume remains unchanged during heating in a mammary carcinoma of mice, it decreases drastically after heating. As a consequence, an increasing number of tumour cells die when the tumours are left *in situ*.

The SCK tumour, a mammary carcinoma of A/J mice, was used. This tumour is non-immunogenic or very weakly immunogenic, and grows well *in vitro* and *in vivo*. Tumours growing in the flank of female mice were excised, minced and trypsinized. About $5 \times 10^4$ cells able to exclude trypan blue were injected s.c. into the leg of female mice. Tumours 7–8 mm in diameter, 8–10 days after the inoculation, were heated and the functional intravascular volume and cell survival assayed.

For the heating, we lightly anaesthetized the animals with pentobarbital (0·04 mg/g) and taped them on a plexiglass board. The board was placed over a 43·5°C waterbath and the tumour-bearing legs were immersed in the water through 2·5cm diameter holes in the board. The intratumour temperature, measured by a 29-gauge thermocouple, rose to 42·9–43·1°C within a few minutes. We heated the tumours for 30 min or 1 h and measured the functional vascular volume using the $^{51}$Cr-labelled red blood cell ($^{51}$Cr-RBC) method as described elsewhere (Song & Levitt, 1971). Briefly, RBC of A/J mice were labelled with [$^{51}$Cr]-$Na_2CrO_4$ and about 0·05 ml of suspension of the labelled RBC was injected into the tail vein of tumour-bearing mice without anaesthesia. Fifteen minutes after the $^{51}$Cr-RBC injection, the mice were lightly anaesthetized with ether and the tumours were excised. Except for heating of tumour, the same procedure including anaesthesia was applied to the control mice. The groin area was cut with scissors and 0·2–0·3 ml of blood was collected in a heparinized syringe. We counted the radioactivity of the tumours and in 0·1 ml of blood obtained from the same animals with a well-type $\gamma$-scintillation counter and calculated the functional intravascular volume using the following formula:

$$\text{Vascular volume} = \frac{(^{51}\text{Cr activity/g of tumour})}{(^{51}\text{Cr activity/ml of blood})}$$

Since the haematocrit of blood from the tumours may be slightly different from the haematocrit of blood from the general circulation, the above-calculated vascular volume may not be exactly the volume of

*Present address: College of Natural Sciences, Seoul National University, Seoul, Korea.

FIG. 1.—Vascular volume of tumours at various times after heating with 43·5°C waterbath for 0·5 and 1·0 h. The intratumour temperature during the heating was 42·9–43·1°C. The vascular volume in control tumours is also shown. The closed circles are values for individual tumours and the open circles are the geometric mean for each group. The bars represent s.e.

circulating blood in the tumours. However, the data obtained with this method may suffice in our attempt to quantitate relative changes in vascular volume in heated tumours.

Fig. 1 shows the change in the functional intravascular volume in SCK tumour. The vascular volume of unheated control tumours was 8·80 ± 1·41 ml/100 g. The vascular volume in tumours at the end of a 1h heating, measured by injecting the $^{51}$Cr-RBC 15 min before the termination of heating and excision of tumours, was 8·47 ± 1·05 ml/100 g. This indicated no significant change in vascular volume or blood perfusion during the 1h heating with a 43·5°C waterbath. However, the vascular volume decreased drastically to 0·37 ± 0·08 ml/100 g and 0·42 ± 0·06 ml/100 g at 7 and 20 h respectively after the heating. Heating the tumours for only 30 min also produced a remarkable reduction in vascular volume; the vascular volume measured 5 and 20 h after heating for 30 min was 0·84 ± 0·15 ml/100 g and 1·23 ± 0·29 ml/100 g respectively. These results clearly demonstrated that the blood perfusion in the SCK tumour decreased to less than 10% of control over several hours after heating for as little as 30 min. The effects of various temperatures and duration of heating on the tumour vasculature remains to be investigated.

The mechanism for the profound decrease in vascular volume in tumours after heating is not clear. Reinhold *et al.* (1978) observed cessation of microcirculation in tumours growing in transparent chambers when heated at 42·5°C for 3 h. Although this study was rather qualitative, the general conclusion appeared to be consistent with ours, that severe vascular damage occurs in tumours after hyperthermia. Von Ardenne (1978) reported that vascular occlusion occurs when mouse tumours are heated after the tumours have been made acidic by infusing large amounts of glucose. Our present study indicates that the infusion of glucose is not necessary to induce vascular damage in tumours by hyperthermia at 43·5°C. Overgaard (1978) reported the presence of hyperaemia, cyanosis, and haemorrhage in heated mouse tumours. The hyperaemia may be caused by obstruction of outflow of blood from the affected area (passive hyperaemia). Histopathological studies indicate that vascular damage may occur also in human tumours after heat treatment (Sugaar & LeVeen, 1979; Storm *et al.*, 1979).

The question then arose whether cells would be able to survive in the heated tumours with the severe vascular damage observed in this study. We therefore measured the survival of clonogenic cells in the tumours, using the *in vitro* assay method at various times after heating. We heated the tumours with a 43·5°C waterbath for 30 min and excised at various times between 0 and 24 h thereafter, and prepared a single-cell suspension as described above. The cells were cultured in Falcon tissue-culture flasks with RPMI 1640 medium supplemented with 10% foetal calf serum, and the clones were stained and counted 8 days later. Fig. 2 shows the survival of clonogenic cells, calculated from the number of cells obtained per gram of tumour times the

FIG. 2.—Change in cell survival in tumours at various times after heating with 43·5°C waterbath for 30 min. The survival of clonogenic cells was calculated from the number of cells obtained from 1 g of tumour tissue × their plating efficiency. The circles indicate the values for individual tumours and the line follows the geometric mean for each group.

plating efficiency of these cells. The number of recoverable cells from the control tumour was $6\cdot2\times10^7$/g, and the plating efficiency was 55–65%. Thus the number of clonogenic cells in control tumours was $3\cdot6\times10^7$/g. The number of clonogenic cells in tumours excised immediately after heating for 30 min was $7\cdot7\times10^6$/g, ~21·4% of control, the decrease being mainly due to the decrease in plating efficiency to 13·2% (22·5% of control). This suggested that the cells had not been lysed, but were unable to form clones when the tumours were excised immediately after heating for 30 min and cultured. When the tumours were left *in situ*, the number of recoverable cells progressively and remarkably decreased until 12 h: $6\cdot1\times10^6$ and $9\cdot3\times10^5$/g at 5 and 12 h respectively. The plating efficiency also decreased from 13·2% immediately after heating to 6·4% at 5 h. Unlike the number of recoverable cells, which began to increase after 12 h, the plating efficiency started to recover after 5 h, and was 17·2% at 12 h. As a result, the cell survival was only 1·1% and 0·44% of controls, respectively, at 5 and 12 h after heat treatment. At 24 h after the heating, cell survival was about 3% of control, the increase being due to a recovery both in cell number and in plating efficiency.

In this study, we expressed our results in terms of the number of clonogenic cells per gram of tumour. This would not be justified if the tumour weight increased on account of oedema or vasodilation after hyperthermia. It should be pointed out that oedema or vascular pooling do not occur in the heated tumour, whereas adjacent normal tissue becomes oedematous upon heating (Song, 1978). In fact, we found that whereas the ratios of wet weight to dry weight were 2·89 and 3·25 in the control and heated mouse skin (43·0°C, 30 min) respectively, they were 6·22 and 6·20 in the control and heated SCK tumour respectively.

Our result is in close agreement with the recent observation by Marmor *et al.* (1979) who reported that when EMT6 tumours of mice were left *in situ* after heating, the cell death progressed, and they concluded that the direct killing of tumour cells by heat was too small to account for the frequent cure of tumours by hyperthermia.

The tumoricidal effect of hyperthermia has been related to an increase in immunological response (Mondovi *et al.*, 1972; Muckle & Dickson, 1971; Sugaar & LeVeen, 1979). However, in view of the fact that the SCK tumour we used is nonimmunogenic, the possibility that the remarkable cell death during the several hours after heat treatment was due to immune mechanisms can be excluded from the present study. An increase in anaerobic glycolysis due to hypoxia as a result of vascular damage would increase the acidity in the heated tumours. Such an increase in acidity may enhance the lysosomal enzyme activity (Overgaard, 1978). We therefore feel that vascular damage is the primary cause of delayed cell death in tumours after hyperthermia. In this connection, Dewey (1979) reported that cell survival decreased dramatically when the cells were left under low pH conditions for a few hours after hyperthermia *in vitro*. An occlusion of vasculature has been reported to enhance thermal sensitization of a mouse tumour, possibly due to an increase in tumour acidity (Hill & Denekamp, 1978). It is conceivable that fractionated hyperthermia may be more effective than a single dose of hyperthermia, if vascular damage can be induced by initial doses of heat during the course of fractionated treatment. In conclusion, the tumoricidal effect of hyperthermia may partly be attributed to the selective destruction of tumour vasculatures and subsequent necrosis in tumours, in addition to the direct cytocidal effect of heat.

This work was supported by NIH Grants CA 13353 and CA 21281.

We thank S. Stettner for skilful and diligent technical assistance, and M. Weiss for preparation of the manuscript.

### REFERENCES

DEWEY, W. C., HOPWOOD, L. E., SAPARETO, S. A. & GERWECK, L. E. (1977) Cellular responses to combinations of hyperthermia and radiation. *Radiology*, **123**, 463.

DEWEY, W. C. (1979) *Cell biology of hyperthermia and radiation*. Presented at the 32nd *Ann. Symp. on Fundamental Cancer Res.-Radiat. Biol. Cancer Res., Houston.*

HAHN, G. M. (1974) Metabolic aspects of the role of hyperthermia in mammalian cell inactivation and their possible relevance to cancer treatment. *Cancer Res.*, **34**, 3117.

HILL, S. A. & DENEKAMP, J. (1978) The effect of vascular occlusion on the thermal sensitization of a mouse tumour. *Br. J. Radiol.*, **51**, 997.

MARMOR, J. B., HILERIO, F. J. & HAHN, G. M. (1979) Tumour eradication and cell survival after localized hyperthermia induced by ultrasound. *Cancer Res.*, **39**, 2166.

MONDOVI, B., SANTORO, A. S., STROM, R., FAIOLA, R. & FANELLI, A. R. (1972) Increased immunogenicity of Ehrlich ascites cells after heat treatment. *Cancer*, **30**, 885.

MUCKLE, D. S. & DICKSON, J. A. (1971) The selective inhibitory effect of hyperthermia on the metabolism and growth of malignant cells. *Br. J. Cancer*, **25**, 771.

OVERGAARD, J. (1978) The effect of local hyperthermia alone, and in combination with radiation, on solid tumors. In *Cancer Therapy by Hyperthermia and Radiation*. Ed. Streffner *et al.* Baltimore: Urban & Schwarzenberg. p. 49.

REINHOLD, H. S., BLACHIEWICZ, B. & BERG-BLOK, A. (1978) Decrease in tumor microcirculation during hyperthermia. In *Cancer Therapy by Hyperthermia and Radiation*. Ed. Streffner *et al.* Baltimore: Urban & Schwarzenberg. p. 231.

SONG, C. W. (1978) Effect of hyperthermia on vascular functions of normal tissues and experimental tumors. *J. Natl Cancer Inst.*, **60**, 711.

SONG, C. W., & LEVITT, S. H. (1971) Quantitative study of vascularity in Walker carcinoma 256. *Cancer Res.*, **31**, 587.

SONG, C. W., RHEE, J. G., LEVITT, S. H. (1979) Blood flow in normal tissues and tumors during hyperthermia. *J. Natl Cancer Inst.*, (in press).

STORM, F. K., HARRISON, W. H., ELLIOTT, R. S., MORTON, D. L. (1979) Normal tissue and solid tumor effects of hyperthermia in animal models and clinical trials. *Cancer Res.*, **39**, 2245.

SUGAAR, S. & LEVEEN, H. H. (1979) A histopathologic study on the effects of radiofrequency thermotherapy on malignant tumors of the lung. *Cancer*, **43**, 767.

VON ARDENNE, M. (1978) On a new physical principle for selective local hyperthermia of tumor tissues. In *Cancer Therapy by Hyperthermia and Radiation*. Ed. Streffner *et al.* Baltimore: Urban & Schwarzenberg. p. 96.